# Different carbohydrate exposures and weight gain—results from a pooled analysis of three population-based studies

**DOI:** 10.1038/s41366-023-01323-3

**Published:** 2023-05-06

**Authors:** Rilla Tammi, Satu Männistö, Kennet Harald, Mirkka Maukonen, Johan G. Eriksson, Pekka Jousilahti, Seppo Koskinen, Niina E. Kaartinen

**Affiliations:** 1 Department of Public Health and Welfare, Finnish Institute for Health and Welfare (THL), Helsinki, Finland; 2 Department of General Practice and Primary Health Care, University of Helsinki and Helsinki University Hospital, Helsinki, Finland; 3 Folkhälsan Research Center, University of Helsinki, Helsinki, Finland; 4 Department of Obstetrics and Gynecology and Human Potential Translational Research Programme, Yong Loo Lin School of Medicine, National University of Singapore, Singapore, Singapore; 5 Singapore Institute for Clinical Sciences (SICS), Agency for Science, Technology and Research, (A*STAR), Singapore, Singapore

**Keywords:** Risk factors, Lifestyle modification, Nutrition, Weight management

## Abstract

**Background:**

The role of carbohydrate quantity and quality in weight gain remains unsolved, and research on carbohydrate subcategories is scarce. We examined total carbohydrates, dietary fiber, total sugar, and sucrose intake in relation to the risk of weight gain in Finnish adults.

**Methods:**

Our data comprised 8327 adults aged 25−70 years in three population-based prospective cohorts. Diet was assessed by a validated food frequency questionnaire and nutrient intakes were calculated utilizing the Finnish Food Composition Database. Anthropometric measurements were collected according to standard protocols. Two-staged pooling was applied to derive relative risks across cohorts for weight gain of at least 5% by exposure variable intake quintiles in a 7-year follow-up. Linear trends were examined based on a Wald test.

**Results:**

No association was observed between intakes of total carbohydrate, dietary fiber, total sugar or sucrose and the risk of weight gain of at least 5%. Yet, total sugar intake had a borderline protective association with the risk of weight gain in participants with obesity (RR 0.63; 95% CI 0.40−1.00 for highest vs. lowest quintile) and sucrose intake in participants with ≥10% decrease in carbohydrate intake during the follow-up (RR 0.78; 95% CI 0.61−1.00) after adjustments for sex, age, baseline weight, education, smoking, physical activity, and energy intake. Further adjustment for fruit consumption strengthened the associations.

**Conclusions:**

Our findings do not support an association between carbohydrate intake and weight gain. However, the results suggested that concurrent changes in carbohydrate intake might be an important determinant of weight change and should be further examined in future studies.

## Introduction

The growing prevalence of obesity is a major public health challenge worldwide. In 2016, globally 1.9 billion adults were living with overweight (body mass index (BMI) ≥ 25 kg/m^2^), and of those, more than 650 million were living with obesity (BMI ≥ 30 kg/m^2^) [1]. The prevalence of obesity reached 42% among American adults in 2017−2018, while in Finland, more than 25% of the adult population was living with obesity in 2017 [2, 3]. Obesity contributes heavily to the development of several noncommunicable diseases, such as type 2 diabetes, cardiovascular diseases, and several cancers [4]. Thus, finding effective ways to tackle the current obesity epidemic is essential in ameliorating public health and decreasing the burden of noncommunicable diseases in populations.

Diet is a central risk factor for obesity, but consensus on the role of different macronutrients in obesity development is lacking. High carbohydrate intake has been suggested to promote weight gain by affecting metabolic processes, such as appetite regulation, independent of energy intake [5, 6]. However, the evidence from randomized controlled trials as well as observational studies has been conflicting, and the association between carbohydrate intake and body weight remains unsolved [7–9]. In a 5-year follow-up of 373,803 adults from ten European countries (EPIC-PANACEA Study), participants in the highest tertile of energy intake from carbohydrates gained less weight than those in the lowest tertile [10]. In contrast, no association was found between carbohydrate intake and weight change in 1762 Danish adults followed for 5 years or in a 4-year follow-up of 465 American adults [11, 12].

Carbohydrate quality has been considered increasingly in examining associations between carbohydrates and health. Especially sugar-sweetened beverages (SSB) have drawn attention, as meta-analyses of randomized controlled trials and cohort studies have provided evidence of their positive associations with weight gain and obesity [13, 14]. Less studies have examined total sugars or sugar subcategories (e.g., sucrose) in this context, and evidence from population-based cohort studies is largely lacking. The association between high sucrose intake and anthropometric measures has been examined only in few prospective studies with inconsistent results [12, 15]. Research on total sugars and sucrose (as a surrogate measure for added sugars) is warranted to elucidate the obesity relations of sugars from the whole diet, including naturally occurring sugars and added sugars from other sources than SSB.

High intake of dietary fiber is generally considered to protect against weight gain [16, 17]. In a 6.5-year follow-up of 89,432 participants from five European countries, intake of dietary fiber was inversely associated with subsequent weight change [18]. Similarly, in a 12-year follow-up of 74,091 participants in the Nurses’ Health Study, the likelihood of major weight gain was lower among those with higher dietary fiber intake compared to those with lower intake [19]. Yet, apart from a study conducted in Denmark applying data from the 1970s and 1980s, prospective studies examining the association between dietary fiber intake and body weight are lacking in Nordic countries [11].

As the role of carbohydrate quantity and quality in weight gain remains unsolved and prospective studies, especially in Nordic countries, are scarce, we aimed to examine carbohydrate intake in relation to subsequent weight change in Finnish adults by pooling three population-based cohort studies. In addition to total carbohydrate intake, the intake of dietary fiber, total sugars, and sucrose were examined.

## Methods

### Participants

The pooled analysis included three population-based follow-up studies: the Health 2000 and 2011 Health Examination Surveys (Health 2000), the clinical examinations of the Helsinki Birth Cohort Study (HBCS) 2001−2004 and 2011−2013, and the Dietary, Lifestyle, and Genetic Determinants of Obesity and the Metabolic Syndrome (DILGOM) Study 2007 and 2014. The Health 2000 Survey aimed at assessing health, functional capacity, and their determinants in Finnish adults [20]. It comprised a nationally representative population-based sample of 8028 adults aged 30 years and over who were selected using a stratified two-stage cluster design and invited to the baseline health examination (Table 1). The HBCS was set up to investigate the effect of early growth on health in later life [21]. The original cohort included individuals born in Helsinki between 1934 and 1944 (*n* = 8760). They were traced again in the year 2000 using the Finnish population register, and a health questionnaire was sent to them resulting in 4515 responses. Of these, 2902 individuals were chosen by random-number tables and invited to a clinical health examination in 2001−2004. The DILGOM baseline study was conducted as a part of the National FINRISK 2007 Study, which comprised a random sample of 10,000 individuals aged 25–74 years drawn from the Finnish population register from five geographical areas [22]. All individuals who participated in the FINRISK 2007 Study (*n* = 6258) were invited to a second, more detailed health examination focused on the determinants of obesity and metabolic syndrome [23]. A detailed description of the study protocols can be found elsewhere [20–22]. All studies included health examinations (at baseline and follow-up) and questionnaires.

**Table 1:** Characteristics of the data included in the pooled analysis.

Characteristics	Health 2000	HBCS	DILGOM^a^
*Baseline*			
Year of data collection	2000	2001−2004	2007
Age range, years	≥30	56−70	25−74
Invited, *n*	8028	2902	6258
Participated, *n* (% of the invited)	6823 (85%)	2003 (69%)	5024 (84%)
*Follow-up*			
Year of data collection	2011	2011−2013	2014
Invited	Participants at baseline^b^	Participants at baseline^b^	Participants at baseline^b^
Participated, *n*	4218	1094	3735
Analytical sample^c^, *n*	3993	1065	3269

*DILGOM* the Dietary, Lifestyle, and Genetic Determinants of Obesity and the Metabolic Syndrome Study; *HBCS* the Helsinki Birth Cohort Study; *Health 2000* the Health 2000 Health Examination Survey

^a^The DILGOM Study was implemented within the framework of the FINRISK 2007 Study. All participants in the FINRISK 2007 Study health examination were invited to the DILGOM 2007 Study.

^b^Participants at baseline excluding deceased, those who had moved away from Finland, and those who had declined further contact.

^c^Exclusion criteria: Inadequately completed or missing FFQ at baseline, missing information on body weight or BMI at baseline or follow-up, age >70 years at baseline, BMI < 18.5 kg/m^2^ at baseline or follow-up, pregnancy at baseline or follow-up.

The studies were conducted according to the guidelines laid down in the Declaration of Helsinki, and all procedures involving human subjects were approved by the Ethics Committee of the Hospital District of Helsinki and Uusimaa. Written informed consent was obtained from all participants.

### Demographic variables, lifestyle variables, and anthropometrics

The age and sex of the participants were derived from the sampling frame. Data on participants’ educational attainment, smoking status, and habitual leisure-time physical activity were gathered by comparable self-administered questionnaires. Educational attainment was determined as total years of education. Smoking status was assessed by three categories (current smoker, former smoker, never smoker). Similarly, leisure-time physical activity was categorized according to a three-level scale (mild shortness of breath and perspiration <1 time/week, 1−3 times/week, ≥4 times/week).

Anthropometric measurements, including weight, height, and waist circumference (WC), were measured in health examinations according to international standard protocols by trained research staff [24, 25]. In the DILGOM follow-up study, 35% of the cohort was invited to participate in the health examination, while the rest provided self-reported measurements by postal questionnaire [26]. BMI (kg/m^2^) was derived by dividing weight (kg) by squared height (m). Normal weight was defined as BMI 18.5−< 25 kg/m^2^, overweight as BMI 25−< 30 kg/m^2^, and obesity as BMI ≥ 30 kg/m^2^ [27].

### Diet

Habitual food intake was assessed in each cohort at baseline and follow-up by a similar, validated semi-quantitative food frequency questionnaire (FFQ) that was slightly updated between the studies [28–30]. The FFQ included queries on the average consumption of 125 food items in the Health 2000, 128 food items in the HBCS, and 131 food items in the DILGOM over the previous 12 months. The food consumption was reported according to nine frequency categories ranging from never or seldom to six or more times a day. Portion sizes were specified in natural units (e.g., slice, glass) for each food item and mixed dish. The FFQ was completed either at the study site or at home. The average daily food consumption (grams/day [g/d]), nutrient (g/d), and energy intake (kilojoules/day [kJ/d]) were calculated by applying an in-house software which utilizes the Finnish Food Composition Database [31].

### Study population

The study population comprised participants with acceptably completed FFQ at baseline and information on body weight and BMI both at baseline and follow-up. We excluded participants older than 70 years at baseline and those with BMI lower than 18.5 kg/m^2^ or pregnant at either time point. Thus, our final analytical sample consisted of 8327 participants (Health 2000 *n* = 3993, HBCS *n* = 1065, DILGOM *n* = 3269) (Table 1).

### Statistical analyses

All statistical analyses were conducted in R version 3.6 [32]. The exposure variables (total carbohydrate [available carbohydrates excluding dietary fibers], dietary fiber, total sugar and sucrose intake) were log (natural)-transformed to meet the assumption of normal distribution. Descriptive data, including means and standard deviations or percentages for the exposure and outcome variables and background factors, were calculated separately for each cohort. The exposure variables were divided into quintiles by study-specific cut-offs. Confounding variables were chosen based on the literature on associations between carbohydrate intake and obesity measurements. We applied three models to adjust for confounders: model 1 (sex, age, weight at baseline), model 2 (model 1 + education, smoking, leisure-time physical activity), and model 3 (model 2 + energy intake). In addition, two sensitivity models were constructed, one to account for naturally occurring sugars from fruits (model 4: model 3 + fruit consumption), and one to account for energy under-reporting (model 5: model 3 excluding energy under-reporters). Fruit consumption was included in the adjustments as fruits are an important source of sucrose in the diet of Finnish adults [33]. Energy under-reporters were identified by calculating the ratio of reported energy intake and predicted basal metabolic rate (BMR) [34]. A cut-off value of 1.14 was applied, a lower value representing under-reporting (EI:BMR ≤ 1.14) [35].

The follow-up times were standardized to 7 years (according to the shortest follow-up in the DILGOM study) by dividing the change in outcome variables during the follow-up by the follow-up time and multiplying it by seven. We applied two-stage pooling to derive relative risks (RR) for weight gain (or increase in waist circumference) of at least 5% according to different exposures in a 7-year follow-up. We examined ≥5% weight change as it has been considered clinically significant [36, 37]. Change in waist circumference was examined as an additional analysis to consider the relevance of weight change for metabolic health. We used RR instead of odds ratio (OR), as RR is commonly used in cohort studies examining health outcomes. Moreover, it is known that OR tends to be interpreted as RR, even though it overestimates risk, especially when the incidence of the outcome is common (>10%), like in this study [38]. To calculate RRs, we used logistic regression with logarithm as the link function. The pooled RRs (and 95% CIs) were calculated with the meta package for R programming language using random effects model and weighting study-specific RRs by the inverse of their variance [39, 40]. The RRs were examined in intake quintiles of the exposure variables. Two-sided *P*-trends across quintiles (exposure quintile medians as continuous variables) were based on a Wald test of the pooled estimates. Two-sided *P*-values for heterogeneity between the study-specific RRs were derived using the Q-statistics.

Using one-stage pooling, we also conducted interaction analyses between the exposures and population subgroups (sex, age, education, smoking, physical activity, baseline BMI, baseline fruit consumption, change in total carbohydrate intake) in the associations of exposure intakes and weight gain of at least 5% in 7 years. HBCS was excluded from the analyses between the subgroups of change in carbohydrate intake as there were too few participants whose carbohydrate intake decreased during the follow-up. Adjustments for multiple comparisons were not made. A two-sided value of *P* < 0.05 was considered statistically significant.

## Results

Fifty-five percent of the participants were women. The mean age at baseline was approximately 50 years in the Health 2000 and DILGOM and 61 years in the HBCS (Table 2). The mean BMI was 27 kg/m^2^ at baseline and the proportion of participants with obesity was ~20% in all studies. The average energy intake at baseline ranged from 9.2 MJ/d (2199 kcal/d) in the HBCS to 10.5 MJ/d (2510 kcal/d) in the DILGOM. Carbohydrate intake varied between 44E% (Health 2000) and 49E% (DILGOM).

**Table 2:** Baseline characteristics (means and standard deviations [SD] or percentages) of the participants in the Health 2000 Survey, the HBCS, and the DILGOM Study.

	Health 2000	HBCS	DILGOM
	Mean (SD) or %	Mean (SD) or %	Mean (SD) or %
Cohort size, *n*	3993	1065	3269
Women, %	55	56	54
Age, years	48 (11)	61 (3)	51 (12)
Education, years	12 (4)	13 (4)	13 (4)
Current smokers, %	27	19	16
Exercise <1 times/wk^a^, %	23	31	17
Weight^b^, kg	77 (15)	78 (14)	76 (15)
Height^c^, cm	169 (9)	169 (9)	169 (9)
BMI, kg/m^2^	26.7 (4.5)	27.3 (4.2)	26.7 (4.7)
BMI ≥ 30 kg/m^2^, %	21	22	19
Waist circumference^d^, cm	92	94	91
Energy under-reporters^e^, %	30	29	21
Energy intake, MJ/d	9.5 (3.2)	9.2 (3.2)	10.5 (3.7)
Carbohydrate^f^, g/d	246 (86)	253 (94)	295 (109)
Carbohydrate^f^, E%	44 (5)	45 (6)	49 (6)
Fiber, g/d	25 (10)	27 (12)	31 (13)
Fiber, g/MJ	2.7 (0.8)	3.0 (1.0)	3.0 (1.0)
Total sugars, g/d	112 (47)	124 (56)	140 (61)
Total sugars, E%	20 (5)	23 (6)	23 (5)
Sucrose, g/d	50 (26)	52 (28)	62 (34)
Sucrose, E%	9 (3)	10 (4)	10 (4)
Fat, g/d	94 (36)	82 (34)	89 (37)
Fat, E%	36 (5)	33 (5)	31 (5)
Protein, g/d	96 (33)	92 (35)	109 (41)
Protein, E%	17 (2)	17 (3)	18 (3)
Fruits, g/d	221 (211)	289 (263)	279 (232)

*BMI* body mass index, *DILGOM* the Dietary, Lifestyle and Genetic determinants of Obesity and Metabolic Syndrome study, *HBCS* Helsinki Birth Cohort Study, *Health 2000* the Health 2000 Health Examination Survey

^a^Leisure-time physical activity, mild shortness of breath and perspiration <1 time/week.

^b^Mean weight in men: Health 2000, 85 kg; HBCS, 86 kg; DILGOM, 84 kg. Mean weight in women: Health 2000, 70 kg; HBCS, 73 kg; DILGOM, 70 kg.

^c^Mean height in men: Health 2000, 177 cm; HBCS, 177 cm; DILGOM, 176 cm. Mean height in women: Health 2000, 163 cm; HBCS, 163 cm; DILGOM, 163 cm.

^d^Mean waist circumference in men: Health 2000, 97 cm; HBCS, 100 cm; DILGOM, 96 cm. Mean waist circumference in women: Health 2000, 87 cm; HBCS, 90 cm; DILGOM, 86 cm.

^e^Energy under-reporters were identified by a Goldberg cut-off value of 1.14 for the ratio between reported energy intake and predicted basal metabolic rate (EI:BMR ≤ 1.14) (34, 35).

^f^Available carbohydrates excluding dietary fibers.

In total, the participants were followed for ~7.2, 10.3, and 11.0 years in the DILGOM, HBCS, and Health 2000, respectively (Table 3). During the total follow-up, participants in the Health 2000 and DILGOM gained weight on average (1.8 and 1.0 kg, respectively), whereas, in the HBCS, the mean weight change was negative (−1.4 kg). Moreover, in the Health 2000 and DILGOM, the proportion of participants who gained at least 5% weight was double compared to the proportion in the HBCS. A larger proportion of women (DILGOM: 60%; HBCS: 44%; Health 2000; 63%) than men (DILGOM: 55%; HBCS: 34%; Health 2000; 57%) gained weight during the follow-up. The mean change in waist circumference was positive in all cohorts (Health 2000: 2.7 cm; HBCS: 1.4 cm; DILGOM: 2.3 cm) and the proportion of participants with at least 5% increase in waist circumference ranged from 29% in the HBCS to 35% in the DILGOM and 37% in the Health 2000 (data not shown).

**Table 3:** Obesity and carbohydrate intake-related follow-up characteristics of the participants in the Health 2000 Survey, the HBCS, and the DILGOM Study.

	Health 2000	HBCS	DILGOM
	Mean (SD) or %	Mean (SD) or %	Mean (SD) or %
**From baseline**			
Follow-up time, years	11.0	10.3	7.2
*Change in weight*			
Cohort size for weight, *n*^a^	3993	1065	3269
Weight change, kg	1.8 (7.3)	−1.4 (5.7)	1.0 (5.8)
Participants who gained weight, %	60	40	58
Participants who gained weight at least 5%, %	34	14	27
Weight gain in participants who gained weight at least 5%, kg	9.1 (5.6)	7.4 (3.5)	7.6 (4.2)
*Change in carbohydrate intake*			
Cohort size for carbohydrate intake^a^, *n*	3162	1052	2913
Change in carbohydrate intake, g/d	−5.8 (89.3)	−21.7 (93.7)	−55.7 (98.8)
Participants who decreased intake^b^, %	42	48	65
Change in participants who decreased intake, g/d	−82 (54)	−91 (66)	−106 (73)
Participants with ≤10% change in intake, %	24	23	19
Change in participants with ≤10% change in intake, g/d	−1 (15)	−2 (14)	−4 (15)
Participants who increased intake^c^, %	35	29	16
Change in participants who increased intake, g/d	84 (66)	80 (65)	86 (72)

^a^Exclusion criteria: Inadequately completed or missing FFQ at baseline, missing information on body weight or BMI at baseline or follow-up, age >70 years at baseline, BMI < 18.5 kg/m^2^ at baseline or follow-up, pregnancy at baseline or follow-up.

^b^Carbohydrate intake decreased >10%.

^c^Carbohydrate intake increased >10%.

The mean carbohydrate intake decreased in each study population during the total follow-up, the decrease ranging from 5.8 g/d in Health 2000 to 55.7 g/d in DILGOM (Table 3). In Health 2000, the mean carbohydrate intake decreased in 42% of the participants, while the corresponding proportions were 48% in the HBCS and 65% in the DILGOM. The proportion of participants who on average increased their carbohydrate intake varied between the studies from 16% (DILGOM) to 35% (Health 2000). The changes in average carbohydrate intake were similar between sexes in the HBCS (−20 g/d) and DILGOM (−55 g/d), whereas in the Health 2000, the mean carbohydrate intake decreased in women (−16 g/d) and increased in men (+7 g/d).

No statistically significant associations were observed between total carbohydrate, dietary fiber, total sugar, or sucrose intake and the risk of weight gain of at least 5% when the follow-up time was standardized to 7 years (Table 4). There was no significant heterogeneity between the studies. A borderline inverse association, however, occurred between total carbohydrate intake and weight gain when fruit consumption was included in the adjustments (model 4; RR 0.81; 95% CI 0.64−1.03 for highest vs. lowest quintile; *P for trend* = 0.055). Yet, this association disappeared when energy under-reporters were excluded from the analysis (model 5). No associations were observed between exposure variables and a ≥ 5% increase in waist circumference (data not shown).

**Table 4:** Pooled relative risks (RR) and 95% confidence intervals (CI) for weight gain of at least 5% in 7-year follow-up according to exposure fifths at baseline.

	Q1	Q2	Q3	Q4	Q5	*P*_trend_^a^	*P*_heterogeneity_^b^
Carbohydrates							
Median, g/d	156	208	249	300	393	–	–
Weight gain ≥ 5%, *n*	431	395	381	351	362	–	–
Model 1^c^	1.00	0.91 (0.81, 1.03)	0.75 (0.43, 1.31)	0.85 (0.73, 0.99)	0.93 (0.82, 1.05)	0.26	0.33
Model 2^d^	1.00	0.94 (0.83, 1.07)	0.78 (0.44, 1.37)	0.90 (0.79, 1.02)	0.98 (0.86, 1.11)	0.61	0.38
Model 3^e^	1.00	0.92 (0.81, 1.05)	0.83 (0.58, 1.20)	0.84 (0.71, 1.00)	0.87 (0.69, 1.11)	0.18	0.54
Fiber							
Median, g/d	14	21	26	32	43	–	–
Weight gain ≥5%, *n*	464	394	361	347	354	–	–
Model 1^c^	1.00	0.84 (0.63, 1.13)	0.84 (0.66, 1.07)	0.85 (0.75, 0.96)	0.91 (0.73, 1.14)	0.39	0.16
Model 2^d^	1.00	0.89 (0.69, 1.14)	0.90 (0.71, 1.13)	0.93 (0.82, 1.05)	1.00 (0.81, 1.24)	0.89	0.16
Model 3^e^	1.00	0.91 (0.74, 1.11)	0.91 (0.74, 1.13)	0.93 (0.81, 1.07)	1.02 (0.79, 1.31)	0.80	0.19
Total sugars							
Median, g/d	66	92	114	143	195	–	–
Weight gain ≥5%, *n*	396	379	386	362	397	–	–
Model 1^c^	1.00	0.90 (0.72, 1.14)	0.95 (0.78, 1.15)	0.89 (0.79, 1.01)	0.98 (0.77, 1.24)	0.75	0.49
Model 2^d^	1.00	0.94 (0.73, 1.20)	1.01 (0.89, 1.16)	0.93 (0.82, 1.05)	1.07 (0.94, 1.21)	0.40	0.76
Model 3^e^	1.00	0.96 (0.77, 1.20)	1.04 (0.91, 1.19)	0.96 (0.83, 1.11)	1.13 (0.94, 1.35)	0.25	0.72
Sucrose							
Median, g/d	23	37	49	65	92	–	–
Weight gain ≥5%, *n*	388	382	389	385	376	–	–
Model 1^c^	1.00	0.92 (0.82, 1.04)	0.94 (0.83, 1.06)	0.92 (0.81, 1.04)	0.93 (0.83, 1.06)	0.40	0.72
Model 2^d^	1.00	0.95 (0.84, 1.08)	0.98 (0.87, 1.11)	0.96 (0.84, 1.08)	0.97 (0.85, 1.10)	0.68	0.84
Model 3^e^	1.00	0.95 (0.84, 1.08)	0.97 (0.85, 1.11)	0.94 (0.82, 1.08)	0.94 (0.80, 1.11)	0.52	0.77

^a^Two-sided *P* value for linear trend across quintiles using exposure quintile medians as continuous variables (Wald test).

^b^Two-sided *P* value for heterogeneity between studies (Q-statistic).

^c^Model 1: adjusted for sex, age, and baseline weight.

^d^Model 2: model 1 further adjusted for education (years), smoking (never, past, current smokers), and leisure time physical activity (low, medium, high).

^e^Model 3: model 2 further adjusted for energy intake (kJ/day, continuous).

Interaction analysis revealed an interaction between BMI groups for the association between total sugar intake and weight gain (*P for interaction* = 0.04) (Supplementary Table S1). Among participants with overweight, total sugar intake was associated with a higher risk of weight gain (model 3; RR 1.36; 95% CI 1.01−1.84), while among those with obesity, the RR suggested a borderline inverse association (model 3; RR 0.63; 95% CI 0.40−1.00). This interaction remained after further adjustment for fruit consumption (*P for interaction* = 0.04) with a strengthened association in participants with obesity, while the association in participants with overweight was no longer significant (data not shown). A significant interaction occurred also between groups of change in carbohydrate intake during the follow-up for the association between sucrose intake and weight gain (*P for interaction* = 0.05, excluding HBCS). Among participants with decreased mean carbohydrate intake, sucrose intake had a borderline protective association with weight gain (model 3; RR 0.78; 95% CI 0.61−1.00), while no association appeared in the other groups. The association between sucrose intake and weight gain in those with decreased mean carbohydrate intake was significant after further adjustment for fruit consumption (model 4; RR 0.76; 95% CI 0.59−0.97). A borderline interaction appeared also between BMI groups in the association of total carbohydrate intake with weight gain (*P for interaction* = 0.05), the association being inverse among participants with obesity (model 3; RR 0.49; 95% CI 0.27−0.89). The association was not significant in other groups. The interaction remained after further adjustment for fruit consumption.

## Discussion

In this pooled analysis of 8327 Finnish adults, no associations were observed between total carbohydrate, dietary fiber, total sugar, or sucrose intake and the risk of weight gain of at least 5% in a 7-year follow-up. Similarly, no association occurred between the exposure variables and a ≥5% increase in waist circumference.

Aligned with our results, no associations between total carbohydrate intake and weight change were detected in previous prospective studies conducted in 1762 Danish and 465 American adults followed for 5 and 4 years, respectively [11, 12]. In contrast, in a study of 373 803 adults from ten European countries (EPIC-PANACEA Study), being in the highest tertile of energy intake from carbohydrates (women: 51E%, men: 49E%) was associated with lower weight gain compared to being in the lowest tertile (women: 38E%, men: 36E%) in a 5-year follow-up (2.1 kg vs. 2.2 kg, respectively) [10].

Overall, the findings on carbohydrate intake and risk of weight gain have been contradictory. The inconsistencies might be linked to the quality of consumed carbohydrates as total carbohydrate intake includes subcategories that may differ in their associations with body weight measures. Furthermore, the major food sources of carbohydrates vary between and within populations. In Finland, the two biggest sources of carbohydrates in the diet are cereals, and vegetables and potatoes [33]. Cereals and vegetables are important sources of dietary fiber, which has been deemed to benefit weight control in randomized controlled trials [16]. However, the third-most important carbohydrate source is beverages, including SSB, which have been positively associated with weight gain [13]. Thus, the combined association of carbohydrate subcategories from different sources with weight gain might be nulled by the opposing effects.

The variation in carbohydrate quality and sources also applies within carbohydrate subcategories. For example, sucrose can originate from fruits and sweetened dairy products as well as confectionery and SSB, and these may associate contrarily with weight gain. Similarly, fiber from cereals, vegetables, or fruits has appeared to associate differently with weight changes. For example, in a cohort of 89 000 European adults, higher intake of cereal fiber was associated with weight loss, while no association was observed between the intake of vegetable or fruit fiber and weight change [18].

Our findings of a protective association between total carbohydrate intake and risk of weight gain among participants with obesity suggest that the associations may differ between BMI categories. A significant interaction was observed between BMI categories also regarding total sugar intake and weight gain with a borderline protective association among participants with obesity. Yet, these findings could have been confounded by energy underreporting or selective misreporting of carbohydrate intake, which are particularly common among participants with overweight or obesity [41]. Regarding total sugar intake, this is supported by the trend toward higher risk of weight gain among participants with normal weight or overweight.

Alike total sugars, there was no statistically significant association between sucrose intake and risk of weight gain in the total study population. We used sucrose intake as an approximation of added sugar intake as Finnish adults consume added sugars mainly as sucrose [42]. Our findings were in line with a previous study conducted in 465 American adults followed for 4 years [12]. In contrast, in the UK, sucrose intake was inversely associated with the risk of overweight or obesity in 1734 participants followed for 3 years [15]. This finding was, however, likely due to misreporting, as a positive association was observed when a biomarker for sucrose was applied. In the systematic review of Te Morenga et al., 11 out of 16 cohort studies with adult participants reported positive associations between dietary sugar intake and obesity measures and the findings from randomized controlled trials supported this result [14]. However, the cohorts and trials were predominantly based on SSB consumption, which have repeatedly been associated with weight gain and obesity [13]. In Finland, consumption of SSB is not as high as in many other Western countries, which may, at least in part, explain why we observed no association in our study population [13].

Carbohydrate intake has been decreasing in Finland over the last decades and a similar trend has occurred, for example, in the USA [43, 44]. This trend may, at least partly, arise from the growing scientific evidence and public awareness of low-quality carbohydrates’ possible adverse health effects [16, 45]. Furthermore, low-carbohydrate diets have become increasingly popular over the last decades, especially in the early 2010s in Finland, affecting the overall perception of carbohydrates’ healthiness [46]. This trend in carbohydrate intake was apparent also in our study sample, the mean change in intake being up to −56 g/d between the baseline and follow-up studies (range of carbohydrate intake at baseline 246−295 g/d, 44−49 E%). For this, we conducted an interaction analysis to examine the possible effect of changes in carbohydrate intake during the follow-up on the association between exposure variables and the risk of weight gain. Changes in carbohydrate intake during the follow-up did not predominantly modify the results. However, a significant interaction occurred regarding sucrose intake, which appeared to be protective against weight gain among participants whose mean carbohydrate intake decreased ≥10 percent during the follow-up. This interaction could arise from the changing share of healthy or unhealthy perceived sucrose sources in a diet (e.g., fruits vs. SSB). Yet, adjusting for fruit consumption did not change the interaction. Overall, alike with carbohydrates in general, more research is warranted on the relevance of different sugar sources in relation to obesity measures.

With significant changes in intake levels, changes are likely to happen also in the quality of consumed carbohydrates. Thus, associations between carbohydrate intake and weight gain could be easier to detect through changing intake levels in the population. So far, studies examining concurrent changes in carbohydrate intake and body weight are scarce. Yet, this approach might provide more insight and consistent results on the dietary factors promoting weight gain in the population [47]. Further research on changes in carbohydrate intake in prospective studies is required to elucidate their role in the development of obesity.

This study has several strengths. We conducted a pooled analysis with three large population-based studies representing the Finnish adult population. The data collection was initiated in the 2000s in all studies, representing well the current food environment. All studies applied comparable methods to gather information on dietary intake, obesity measures, and comprehensive background data. We applied continuously updated FFQ that has been repeatedly validated in the Finnish adult population and found to assess carbohydrate intake acceptably regarding epidemiological research purposes [30]. In addition to total carbohydrate intake, we included different carbohydrate subcategories, thus considering the role of carbohydrate quality along with quantity in our analyses. As dietary intake (and anthropometric measures) was examined both at baseline and follow-up, we were able to consider the changes in carbohydrate intake between the measuring points in our analyses. We adjusted the analyses for several confounding variables.

The study is also subject to limitations. We applied self-reported dietary intake data, which exposes the results to misreporting. However, the exclusion of energy under-reporters did not change our results. On the other hand, the applied cut-off for energy under-reporters might not capture selective misreporting of carbohydrate-containing foods. Despite the several models applied to adjust the analyses for confounders, residual confounding may remain, as body weight and dietary intake are affected by numerous factors that cannot be (fully) adjusted for. Among others, these include genetic traits and microbiome of which significance in obesity development has been increasingly explored over the past years. The key confounding factors were, however, considered in our analyses. We applied 5% as the cut-off for major weight gain due to the limited number of participants with significant weight gain in the HBCS. Yet, 5% might not be substantial enough change to display associations between carbohydrate intake and major weight gain. Nonetheless, the results did not significantly change when a 10% cut-off was applied in data excluding the HBCS (data not shown). As the participants’ weight was measured only at two time points, we could not consider weight fluctuation that may have occurred during the follow-up. Weight fluctuation, however, has been shown to predict larger increases in BMI and waist circumference over time [48]. Thus, unmeasured fluctuation more likely leads to underestimation than overestimation of the risk of weight gain in our study population.

In conclusion, we observed no association between total carbohydrate, dietary fiber, total sugar or sucrose intake and weight gain of at least 5% in a 7-year follow-up of 8327 Finnish adults. As the findings have been, in general, highly inconsistent, further research is warranted from different populations and with modern data to elucidate the relation of carbohydrate quantity and quality with weight gain. Moreover, changes in diet during follow-up should be considered more widely in subsequent studies.

## Supplementary information

Supplemental material

## Data Availability

The datasets analyzed during the current study are available upon request through the Findata permit procedure (https://www.findata.fi/en/).
