# Supplementary material for: Different carbohydrate exposures and weight gain—results from a pooled analysis of three population-based studies
Source: Int J Obes (Lond). 2023 May 6;47(8):743–9. doi: 10.1038/s41366-023-01323-3 (PMC10359185; doi:10.1038/s41366-023-01323-3)
Supplement: Supplementary file 1 — Supplemental material [file 41366_2023_1323_MOESM1_ESM.pdf]

Supplementary Information

International Journal of Obesity

Different carbohydrate exposures and weight gain – results from a pooled analysis of three population-based studies

Rilla Tammi, Satu Männistö, Kennet Harald, Mirkka Maukonen, Johan G. Eriksson, Pekka Jousilahti, Seppo Koskinen, Niina E. Kaartinen

Corresponding author: Rilla Tammi, rilla.tammi@thl.fi

This file includes one supplementary table describing the pooled relative risks for a weight gain of at least 5% in 7-year follow-up by exposure variable in different population subcategories.

Supplementary table S1. Pooled relative risks[1] (RR) and 95% confidence intervals (CI) for a weight gain of at least 5% in 7-year follow-up according to exposure variables in population subcategories.

	Carbohydrate		Fibre		Total sugars		Sucrose	
	RR (95% CI) Q5 vs. Q1	P_{int}[2]	RR (95% CI) Q5 vs. Q1	P_{int}[2]	RR (95% CI) Q5 vs. Q1	P_{int}[2]	RR (95% CI) Q5 vs. Q1	P_{int}[2]
Sex								
Men	0.72 (0.50, 1.05)	0.30	0.95 (0.59, 1.54)	0.11	0.88 (0.65, 1.20)	0.38	0.80 (0.61, 1.05)	0.42
Women	0.98 (0.72, 1.34)		1.07 (0.87, 1.33)		1.28 (1.02, 1.61)		1.03 (0.83, 1.27)	
Age, years								
<50	0.94 (0.70, 1.25)	0.15	0.85 (0.67, 1.08)	0.93	1.36 (0.79, 2.35)	0.28	1.05 (0.76, 1.46)	0.15
≥50	0.78 (0.52, 1.16)		0.99 (0.70, 1.39)		0.92 (0.67, 1.27)		0.93 (0.63, 1.37)	
Education, years								
<12	0.85 (0.58, 1.23)	0.97	1.11 (0.84, 1.47)	0.15	1.12 (0.83, 1.51)	0.35	1.01 (0.77, 1.32)	0.22
≥12	0.89 (0.65, 1.21)		0.95 (0.73, 1.25)		1.11 (0.88, 1.39)		0.88 (0.71, 1.08)	
Smoking								
Never	0.90 (0.64, 1.27)	0.69	0.97 (0.75, 1.24)	0.53	1.15 (0.88, 1.50)	0.68	0.86 (0.67, 1.11)	0.87
Past	0.89 (0.54, 1.48)		0.92 (0.46, 1.82)		1.29 (0.87, 1.91)		1.16 (0.81, 1.66)	
Current	0.79 (0.52, 1.22)		1.25 (0.74, 2.11)		0.99 (0.73, 1.36)		0.89 (0.67, 1.19)	
Physical activity[3]								
Low	0.71 (0.44, 1.14)	0.10	1.04 (0.54, 2.02)	0.48	0.86 (0.60, 1.22)	0.05	0.75 (0.55, 1.02)	0.16
Medium	0.86 (0.58, 1.27)		1.00 (0.79, 1.27)		1.17 (0.91, 1.51)		1.03 (0.82, 1.31)	
High	1.13 (0.70, 1.84)		0.94 (0.60, 1.49)		1.55 (0.93, 2.58)		1.05 (0.69, 1.60)	
Baseline BMI, kg/m^2								
<25	0.78 (0.54, 1.12)	0.05	0.90 (0.70, 1.15)	0.54	1.00 (0.46, 2.16)	0.04	0.99 (0.77, 1.25)	0.07
25-<30	1.21 (0.78, 1.88)		1.23 (0.74, 2.03)		1.36 (1.01, 1.84)		0.99 (0.68, 1.44)	
≥30	0.49 (0.26, 0.89)		0.93 (0.62, 1.42)		0.63 (0.40, 1.00)		0.70 (0.45, 1.07)	

Baseline fruit intake, g/d								
<Median	0.91 (0.62, 1.33)	0.68	1.41 (0.48, 4.15)	0.62	1.03 (0.75, 1.41)	0.36	0.99 (0.75, 1.31)	0.57
≥Median	0.81 (0.58, 1.13)		0.84 (0.51, 1.41)		1.27 (0.95, 1.70)		0.85 (0.65, 1.10)	
Total carbohydrate intake[4], g/d								
Intake increased >10%	1.76 (0.93, 3.33)	0.13	1.26 (0.78, 2.02)	0.63	1.57 (0.94, 2.62)	0.26	1.46 (0.97, 2.20)	0.05
Intake changed ≤10%	0.87 (0.56, 1.34)		1.06 (0.77, 1.45)		1.28 (0.62, 2.65)		0.91 (0.67, 1.22)	
Intake decreased >10%	0.68 (0.48, 0.96)		0.90 (0.56, 1.44)		0.99 (0.67, 1.45)		0.78 (0.61, 1.00)	

BMI, body mass index

[1] Relative risk derived by one-stage pooling.

[2] Two-sided P-value for interaction for the differences in P-trends between population subcategories. Adjusted for sex, age, baseline weight, education (years), smoking (never, past, current smokers), and leisure time physical activity (low, medium, high) and energy intake (kJ/day, continuous).

[3] Leisure-time physical activity, mild shortness of breath and perspiration <1 time/week (low), 1−3 times/week (medium), ≥4 times/week (high)

[4] the Helsinki Birth Cohort Study excluded from the analysis due to a limited number of participants with decreased carbohydrate intake
